# New design for control cage to enhance coverage and uniformity of shot blasting and its validation using DEM and experiment

**DOI:** 10.1038/s41598-023-32563-y

**Published:** 2023-03-30

**Authors:** Hyeryeon Seo, Taehyung Kim, Chulho Yang, Junyoung Park

**Affiliations:** 1 Department of Mechanical Design Engineering, Kumoh National Institute of Technology, 61 Daehak-Ro, Gumi, Gyeongbuk 39177 South Korea; 2 Department of Aeronautic, Mechanical and Electrical Convergence Engineering, Kumoh National Institute of Technology, 61, Daehak-Ro, Gumi, Gyeongbuk 39177 South Korea; 3 Department of Aerospace and Mechanical Engineering, Cheongju University, 298 Daeseong-Ro, Cheongju, Chungbuk 28503 South Korea; 4 Department of Mechanical Engineering Technology, Oklahoma State University, 570A Engineering North, Stillwater, OK 74078 USA

**Keywords:** Mechanical engineering, Theory and computation

## Abstract

Unlike shot peening, shot blasting is a process that primarily uses shot balls to remove foreign substances from metal surfaces. Shot blasting is classified into air-blowing and impeller-impact types. The latter is widely used in commercial large-scale shot blasting. This study proposes a new control cage with a concave or convex shape to improve coverage and uniformity in the impeller-impact type shot blaster. The effectiveness of the proposed control cage is verified using discrete element methods and experiments. Moreover, the optimal design in terms of mass flow, coverage, and uniformity is confirmed. Additionally, the distribution of marks on the surface is analyzed through experiments and simulations. Further, the shot ball is projected over a wider area on the surface when the new concave and convex model is employed at the control cage. Consequently, we confirm that the control cage with a concave shape forms approximately a 5-% higher coverage than the conventional model and uniform shot marks while using a low mass flow rate.

## Introduction

Since the 1950s, the surface treatment and machining of metal using a shot ball have been actively investigated^1–6^. In the steel industry, shot blasting is actively used to prevent deterioration of surface quality by removing foreign substances such as scale on the metal surface of stainless steel. Based on the material and projection method of the shot ball, shot blasting can be classified into air blowing and impeller types. Both shot blasting and shot peening are metal surface treatment processing methods. However, they are classified according to their functions. Shot blasting is used to project shot balls of a metal material onto a surface to remove foreign substances or improve surface roughness by removing the sharp edges of a product. Conversely, shot peening is a machining method for generating residual stress on a surface by projecting shot balls at high speed and increasing surface strength and fatigue life.

The mechanical shot blaster is composed of the distributor, control cage, and blade. First, when the shot ball is supplied inside the rotating distributor, the shot ball exits out of the blaster through a hole in the control cage. And shot blasting is processed in a way that the blade rotating at high speed collides with the shot ball and the shot ball is projected onto the surface. After the shot blasting is processed, coverage and uniformity indicating the processing efficiency are measured. Coverage can be calculated as the ratio of the sum of the shot mark area of the total surface area. Also, uniformity is also measured, indicating how evenly the shot balls are distributed on the surface. The coverage and uniformity are continuously improving, but they still need to be improved further.

The computational approach for shot peening usually focuses on residual stress and primarily adopts a finite element method (FEM). Tange and Okada^7^ repeated such a simulation until 100-% coverage was reached using randomly generated shot balls. They established the relationship between coverage and fatigue strength through experiments and a finite element method. However, under the assumption that all shot marks have the same shape, shot marks that struck the same position were excluded from the calculation of the coverage. Meguid et al.^8^ analyzed the residual stress according to the shape of the shot ball, impact speed, and work hardening of the substrate. As in the previous study, repeated impacts were excluded. Gangaraj et al.^9^ predicted the overall coverage using an axisymmetric model under the assumption of a uniform shot size distribution and a uniform shot mark. Meguid et al.^10^ and Schwarzer et al.^11^ measured the change in residual compressive stress according to the size and speed of a shot ball and measured coverage by periodically repeating a rather unrealistic hexagonal shot mark. Bagherifard et al.^12^ used the same assumption but calculated coverage considering the bounce of a shot ball. Nguyen et al.^13^ showed that coverage is significantly dependent on the size and peening angle of a shot ball in an air-blowing type shot peening through the coupled analysis of CFD and finite element method. Kirk and Abyaneh^14^ numerically calculated coverage by randomly placing shot balls. Taro et al.^15^ succeeded in this technique under the assumption of the same shot size. Marini et al.^16^ investigated the effect of the size and speed of shot balls on surface roughness and residual stress in a micro-shot peening process using finite element method and radiation penetration methods.

As numerous particles contact cannot be utilized in a finite element method, most studies have analyzed with only a few particles^7–16^. When calculating the contact between multiple particles using a finite element method, each particle is discretized with multiple meshes and analyzed. Therefore, using numerous particle contacts in a finite element method is time-consuming. However, in the case of shot blasting or shot peening where coverage and uniformity are more important than residual stress, many shot balls should be used. Therefore, analyzing shot peening and shot blasting using only a finite element method has a limitation.

To address the limitations of the aforementioned studies, many studies have been conducted recently using a discrete element method (DEM). Bhuvaraghan et al.^17^ accurately predicted residual stress and plastic strain using both a finite element method and DEM. Consequently, the residual stress was accurately measured by applying a contact force calculated using the DEM to the finite element method model. Similarly, Hong et al.^18^ used a finite element method and DEM together to intensively analyze the impact of a shot ball that bounced back after hitting the metal surface once. They determined the parameters that should be carefully controlled the most for quality control during shot peening and those that have the greatest influence on residual stress. Murugaratnam et al.^19^ implemented a new algorithm to significantly adjust the coefficient of restitution for the impact of a shot ball repeated at the same point in a DEM. Additionally, the combined effect of the initial velocity, mass flow rate, and pinning angle on the compressive residual stress was analyzed. Hou et al.^20^ analyzed the dynamic behavior of shot balls inside a shot-blasting machine using a 3D DEM. They observed that the faster the rotation speed of the impeller, the greater the rate of change in the speed of the shot ball. Ahmad et al.^21^ calculated the induced compressive stress accurately through a DEM/FEM coupled analysis for the Johnson–Cook material model. In addition, Marini et al.^22^ showed that the measured residual stress field at the V-edge notch agrees well with the finite element method thermal field corrected with experimental data. Further, Choi et al.^23^ proposed a newly designed impeller blade to improve coverage and uniformity through DEMs and experiments.

Similar to analyzing collisions between metal surfaces and shot balls in shot blasting, Dong et al.^24^ proposed a numerical model based on an SPH method to model and simulate the impact process of droplets on elastic beams. In addition, the continuum surface force (CSF) method was adopted to simulate the surface tension effect caused by the impact of the droplet. Droplet impact is mostly observed in ink-jet printing, anti-icing, and pesticide spraying. Van Dam and Le Clerc^25^ experimentally investigated the impact of ink-jet printed droplets on a solid substrate to measure the shape of the impact interface. In addition, the volume of small bubbles of water droplets was experimentally measured in the early stages of the impact and compared with the results using equations. Zhang et al.^26^ conducted a shot-blasting investigation using an SPH method to simulate the impact of particles on a metal surface covered with a rust layer. Further, the impact behavior of particles with different shapes for the deformation and damage of a rust layer was analyzed according to various initial conditions and impact angles.

However, despite the considerable number of related studies, few studies on the control cage, which is one of the basic parts of the impeller-type shot blaster, have been reported regardless of experiments or simulations. In particular, the design of the control cage has thus far been unchanged since the 1960s, implying that the relationship between the design of the control cage and the coverage or uniformity has not been further investigated^27,28^. Therefore, this study showed that the newly proposed control cage design could improve coverage and uniformity using DEM simulation considering multiple particles and collisions between particles. Moreover, the experimental results and simulation were consistent.

## Methods

### Simulation modeling procedure

#### Simulation modeling geometry and parameter

**Figure 1:**
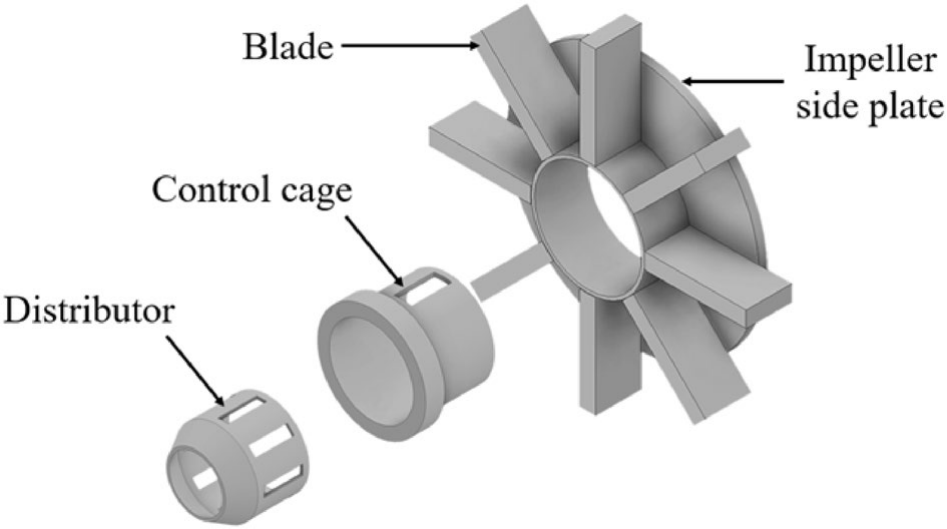
Schematics of impeller-type shot blaster (Inventor 2021, https://www.autodesk.com/products/inventor).

In this study, the impeller-type shot blasting process used in steel companies to remove scale during an iron-making process is analyzed. The shot blaster used in this study consists of a distributor that produces a certain number of shot balls, a control cage that adjusts the direction of the shot, and a blade that accelerates the shot ball, as shown in Fig. 1.

**Figure 2:**
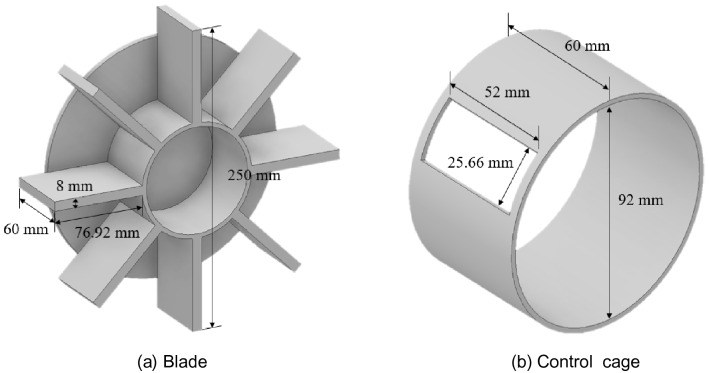
Size of impeller-type shot blaster (Inventor 2021, https://www.autodesk.com/products/inventor).

As shown in Fig. 2a, the blade size used in the simulation is 0.07692 m × 0.06 m × 0.008 m, and the rotation speeds of the distributor and blade are set to 3500 rpm. The overall size of the shot blaster including eight blades is 0.25 m × 0.25 m × 0.07 m, the control cage is inclined 30$^\circ$ clockwise from the horizontal to properly maintain the shot projection angle, and the inner diameter and height are 0.092 and 0.06 m, respectively. As shown in Fig. 2b, the size of the hole in the control cage is 0.052 m × 0.02566 m. The number of shot balls projected by the blaster depends on the shape and size of the hole in the control cage. As the size of the hole decreases, the number of shot balls projected decreases and vice versa. Therefore, the hole shape and the size of the control cage are important parameters for minimizing costs in shot blasting processing.

As shown in Fig. 2, as the substrate moves at 1 m/s in the horizontal direction, the horizontal length is assumed to be infinite and the vertical length is 0.9 m.

The diameter of the shot ball is 0.0008 m, which is the same as the diameter of the shot ball used in the actual machining site. In addition, a general stainless-steel material with a density of 7800 kg/m^3^ and an elastic modulus of 182 GPa is used for both the shot ball and the surface of the substrate. Before the blaster starts rotating, 100,000 shot balls are generated and settle down in the distributor. The blaster rotates counterclockwise at a constant speed and subsequently generated 1.1 kg (approximately 1.6 million) of shot balls per second. All data were extracted and calculated once every 0.01 s from the time of entering the steady state. This study was conducted in EDEM Academic ver. 2021 of Altair, a commercial DEM software. Approximately 96 h is required to calculate the process from the particle generating time to 2 s using the i7-8700 CPU.

#### Evaluating coverage in the simulation model

**Figure 3:**
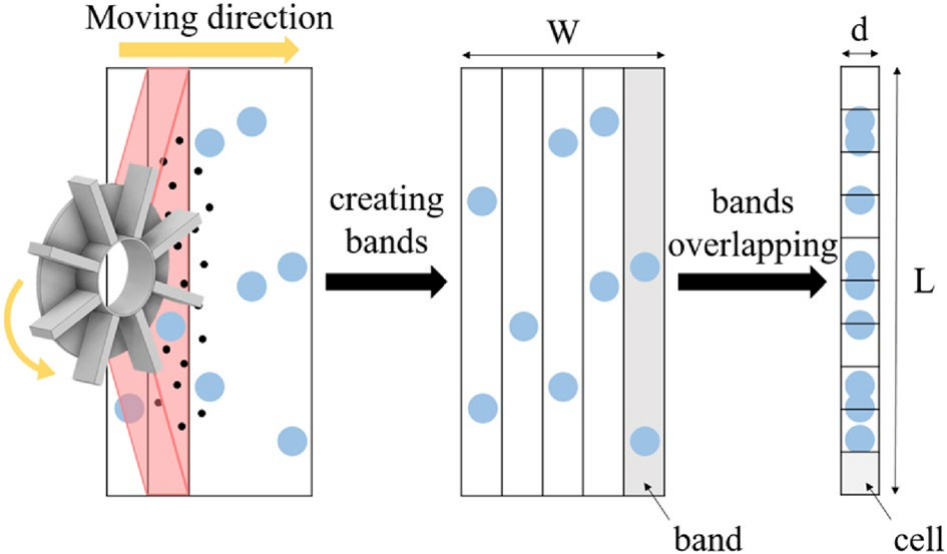
Superimposed shot marks for calculating total coverage (Inventor 2021, https://www.autodesk.com/products/inventor).

In the shot blasting process of the steel company, the blaster is fixed and the steel plate moves continuously in one direction. Accordingly, part of the iron plate is struck several times by shot balls fired from several blades, thereby increasing the coverage. However, if all these processes are analyzed using a DEM, the number of shot balls increases exponentially, rendering the analysis challenging. Therefore, in this study, when calculating coverage, a wide substrate surface is divided into 300 bands (corresponding to 300 blades) at regular intervals in the x-axis direction and overlaps, as shown in Fig. 3. First, shot blasting is performed in an area of *L* × *W*, then divided into 300 bands, and finally superimposed on one band (*L* × *d*). This corresponds to a band size of *L* × *d* shot 300 times.

The shot mark area of each ball, ${A}_{s.m}$ can be calculated through the radius *R* of the shot ball and the maximum normal overlap distance, *δ*_*n,max*_ as follows:

1 $$Are{a}_{a\;shot\;mark} ({A}_{s.m})=\pi \left(2R{\delta }_{n, max}-{\delta }_{n, max}^{2}\right) \left({mm}^{2}\right).$$

**Figure 4:**
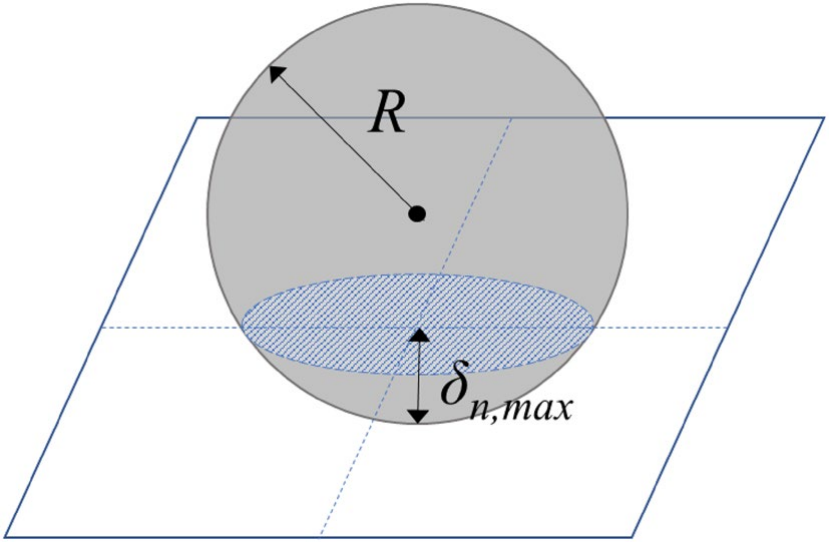
The maximum normal overlap distance of the shot ball and target surface.

Figure 4 shows the radius of the shot ball and the maximum normal overlap distance that occurs between the shot ball and the target surface. Discrete Element Method calculates the force acting on each particle using the distance between particles/particles or between particles/walls. Also, overlap can be easily calculated by subtracting the distance between the particles from the sum of the radii of the two particles.

The total coverage was calculated by dividing the sum of the shot mark areas considering the overlapped area by one band area as follows:

2 $$Coverage \left(C\right)=\frac{\sum A}{Superimposed\;area}=\frac{\sum \pi \left(2R{\delta }_{n,max}-{\delta }_{n,max}^{2}\right)}{Ld} \left(\%\right),$$

where *C* represents the coverage, *L* × *d* is the superimposed area, which is the area of one band, and *A* represents the area of a shot mark.

#### Evaluating uniformity in the simulation model

The uniformity, which shows the extent to which the shot marks by the shot balls are evenly distributed, is an important parameter in exhibiting the quality, economy, and efficiency of a shot blasting process. Therefore, to verify the distribution of shot marks by sections, the overlapped bands should be divided into several cells at regular intervals in the y-direction, as shown in Fig. 3, and the uniformity should be calculated using the difference between the coverage of each of these cells and the total coverage. In other words, the standard deviation of the coverage of each cell is defined as uniformity. A detailed calculation of uniformity is presented in the literature^23^. In this study, uniformity was calculated by dividing one band into 10 cells at regular intervals. A uniformity value close to zero implies that the shot marks are evenly distributed on the surface, and a higher uniformity value indicates that the shot ball marks are concentrated in a single position. Therefore, if the shot marks are uniform everywhere, uniformity approaches zero, and the value increases as the shot marks become non-uniform.

### Proposed design

When shot blasting is performed using a conventional control cage, the distribution of shot marks on the substrate is elliptical. Some studies have reported that the density of shot marks is high in the central area and decreases toward the edges^23^. These elliptical-shaped shot marks have lower coverage and uniformity than rectangular shot marks and, in particular, become more significant as blasting is repeated. To address this issue, we changed the design of the proposed control cage. Notably, this issue can also be addressed by changing the shape of the blade, as reported in the literature^23^.

**Figure 5:**
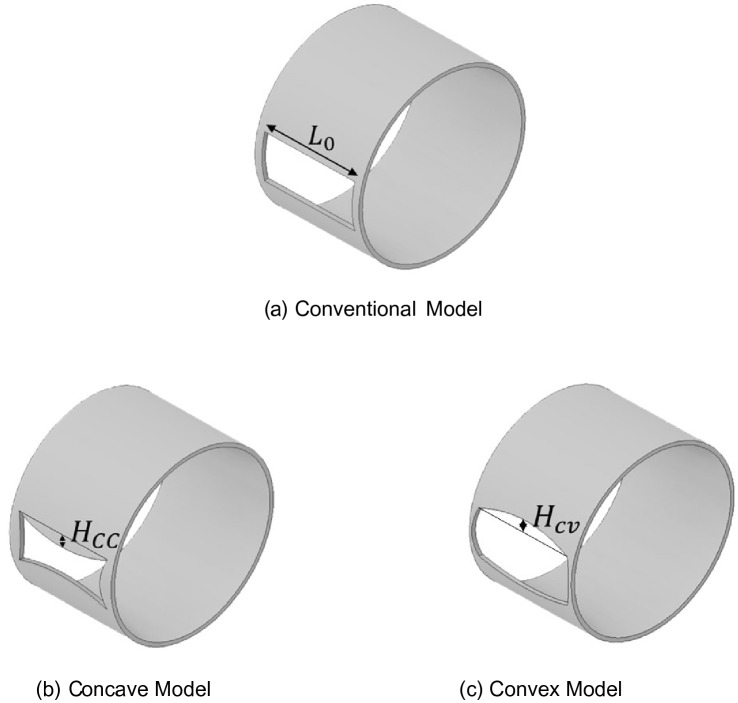
Proposed design of control cage (Inventor 2021, https://www.autodesk.com/products/inventor).

To improve coverage and uniformity, we propose two new shapes of the control cage hole, as shown in Fig. 5. The conventional control cage with a rectangular hole, 0.052 m × 0.033 m, shown in Fig. 5a, is termed the Conventional Model. The modified models are termed the Concave Model, in which the four sides of the hole are concave inward, as shown in Fig. 5b, and the Convex Model, in which the four sides of the hole are convex outward, as shown in Fig. 5c. To measure the degree of concavity and convexity, the ConCave Ratio (CCR) and ConVex Ratio (CVR) are defined as follows:

3 $$Concave\;Ratio \left(CCR\right)= \frac{Concaved\;height\;of\;hole}{Original\;length\;of\;hole}=\frac{{H}_{CC}}{{L}_{o}} \left(\%\right),$$

and

4 $$Convex\;Ratio \left(CVR\right)= \frac{Convexed\;height\;of\;hole}{Original\;length\;of\;hole}=\frac{{H}_{CV}}{{L}_{o}} \left(\%\right),$$

where *L*_*0*_ corresponds to the length (0.052 m) of the hole of the conventional control cage, *H*_*CC*_ is the concave height, and *H*_*CV*_ is the convex height. These ratios were used to determine the degree of concavity and convexity of the horizontal and vertical lengths. As shown in Fig. 5b, the Concave Model consists of four curved surfaces concave inward with heights corresponding to CCR = 5, 10, and 15%. Next, as shown in Fig. 5c, the Convex Model consists of four curved surfaces convex outward with heights corresponding to CVR = 5, 10, and 15%. Therefore, as the CCR increases in the order of 5, 10, and 15%, the Concave Model exhibits more significant concave curved surfaces. Therefore, the area of the hole through which the shot balls exit is reduced. Conversely, the Convex Model exhibits more significant convex shapes as the CVR increases. Therefore, the area through which the shot balls can escape increases.

### Experimental set-up

#### Shot blasting testing machine and specimen

**Figure 6:**
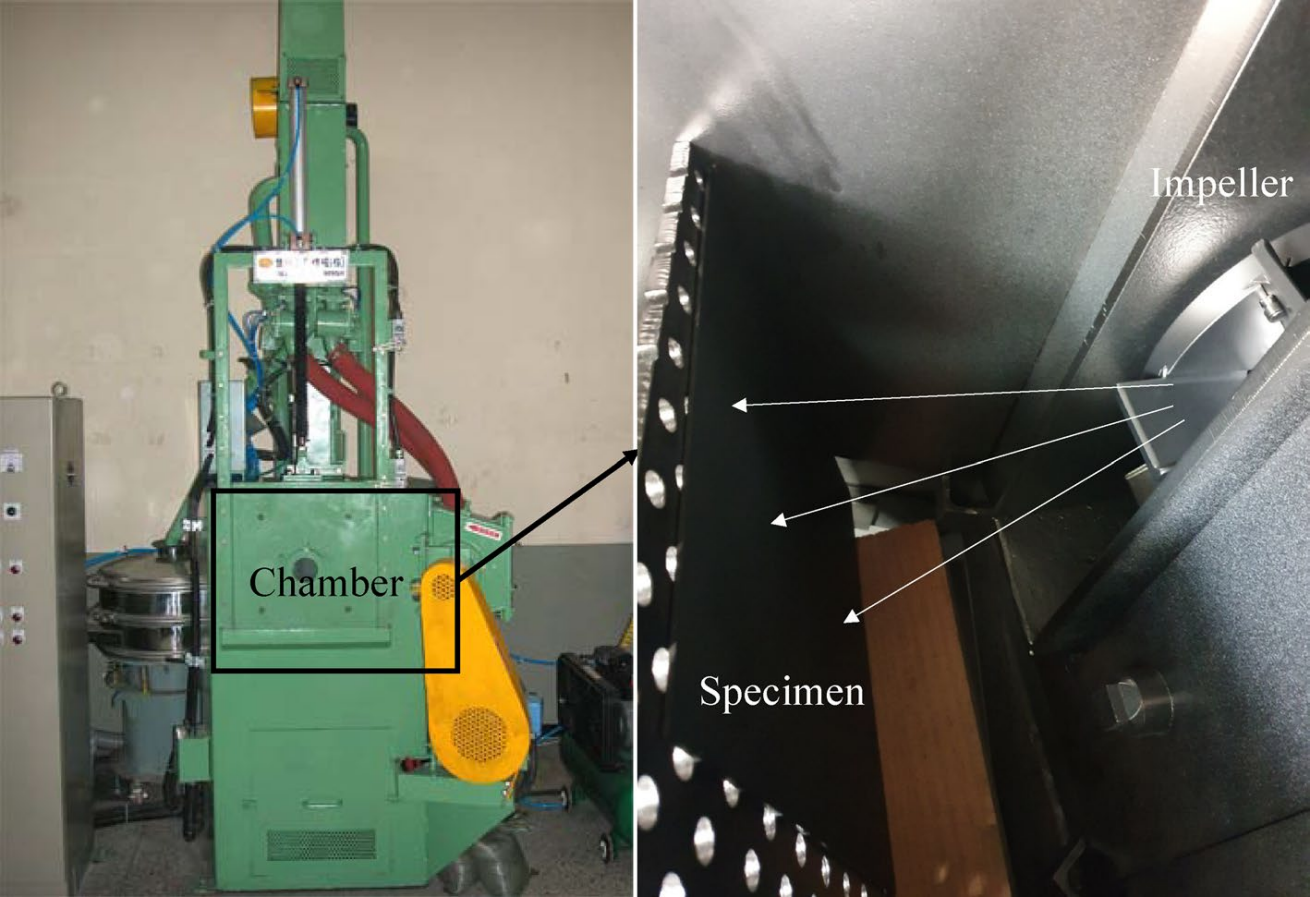
Impeller-typed shot blaster and specimens^23^.

As shown in Fig. 6, shot blasting equipment, similar to that used in the literature^23^, with a 0.25 m diameter impeller (Model No. PMI0608) was used for the experiments. For the steel ball used in the equipment, a cut-wire-rounded shot manufactured by cutting a hard steel wire to a certain length and spheroidizing was used. The average diameter was 0.0008 m. Table 1 shows the chemical composition of the shot balls. The specimen used to measure the coverage of shot blasting was made of SM45C, general industrial carbon steel, with dimensions of 0.3 m in width, 0.3 m in length, and 0.005 m in thickness. SM45C is an alloy of iron and carbon commonly used in general mechanical components. The carbon content is approximately 0.45%. And Table 2 shows the mass flow rate of the shot ball used, the rotation speed of the blade, the Young’s modulus of the shot ball and the target surface, and the number of blades.

**Table 1:** Nominal chemical composition of the shot balls^23^.

Elements	C	Si	Mn	P	S
SWRH 72A (% by weight)	0.69–0.76	0.15–0.35	0.30–0.60	Max. 0.03	Max. 0.03

**Table 2:** The mechanical-related parameters affecting the projectile collision process.

Parameters	Value
Mass rate of shot balls	0.7 kg/s
Blade rotation speed	3500 rpm
Shot ball, target surface Young’s modulus	182 GPa
Number of blades	8

#### Experimental procedure

First, the control cage with a square hole is fastened to the impeller assembly of the blasting equipment. Then, the experiments are performed by rotating the impeller to supply the shot balls. The rotation speed of the impeller is set to 2527 rpm, and the mass of the shot ball supplied to the impeller is 0.4 kg/s. The processing time is set to 5 s, and the surface of the specimen is painted in matte black to easily identified the area where the steel balls collided with the surface of the specimen. After completing shot blasting, the coverage on the specimen surface is measured.

**Figure 7:**
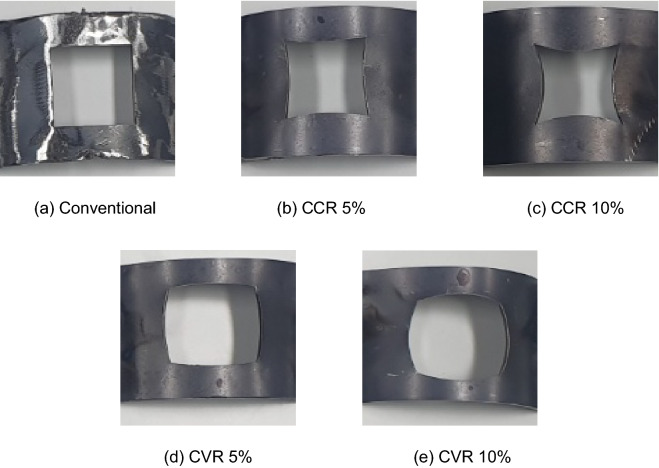
Control cage with various shapes of hole.

Figure 7 shows photographs of the control cages with various hole shapes used in the experiment. All the experiments were conducted by patching a curved plate on top of the existing control cage, as shown in Fig. 7. Notably, the control cage of the blasting equipment cannot easily be changed directly into various shapes during the experiments. Therefore, patches that were slightly smaller than the actual control cage were used. Notably, the hole of the conventional control cage model, which is the standard, has a square shape with a width of 0.0333 m and a length of 0.0333 m. In addition, to realize the shape of various holes, a CCR model concaved with 5% and 10% inward ratios and a CVR model with 5% and 10% outward ratios based on the length of one side were fabricated. By using these control cages, the coverage changes in the specimen surface according to the concavity or convexity were measured. However, we could not manufacture the CCR model concaved at a rate of 15%. Therefore, it was removed from the experiment.

## Results and discussion

### Dynamic behavior of shot ball near control cage

Several studies on shot blasting for metal surface treatment have been reported. Kennedy et al.^29^ investigated micro-shot blasting of machine tools to improve surface finish. The productivity improvement and surface finish improvement methods of the workpiece were explained in the micro blasting of cutting tips and tools. Further, the positive effects of the microblasting of cutting tips were explained by analyzing toughness, life, hardness, and roughness. Jizhan Wu et al.^30^ analyzed the effect of shot-peening coverage on the hardness, residual stress, and surface shape of a carburized roller. Changes in the surface roughness, microhardness, and microstructure of the roller according to the coverage of shot peening were analyzed experimentally.

**Figure 8:**
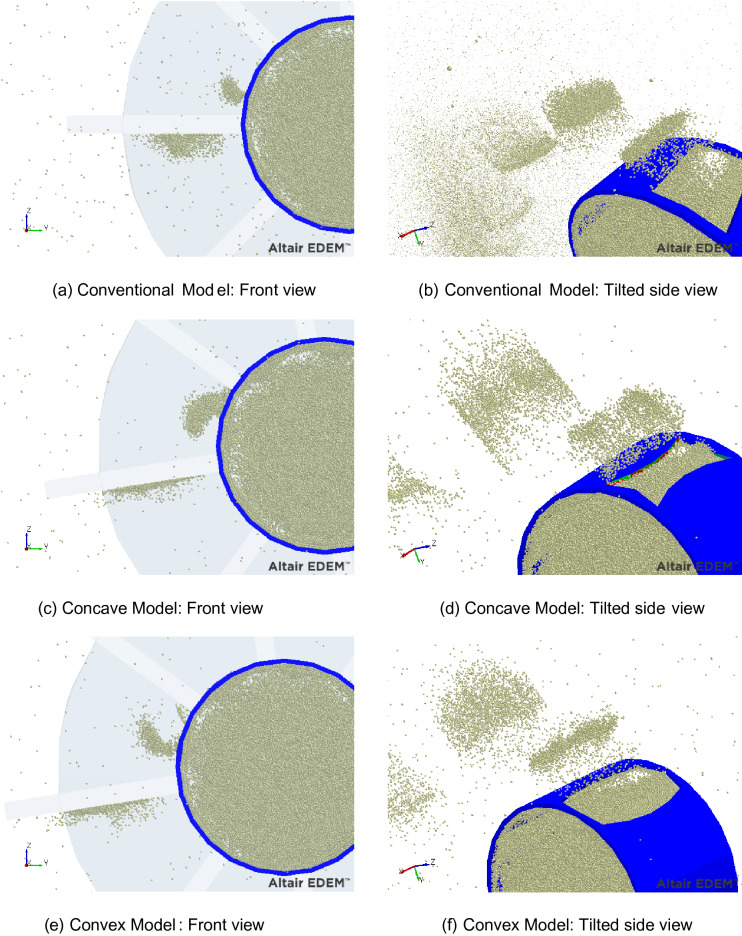
Spatial distribution of shot ball near control cage (Left column: before impact by blade and Right column: after impact by blade, deleted for easy view) (EDEM Academic ver. 2021, https://www.altair.co.kr/edem/).

Generally, during shot blasting, a certain number of shot balls are projected from the distributor and control cage at a given mass. The discharged mass consisting of shot balls is pressed against the blade rotating at a constant speed and thus colliding. The shot balls slid through the blade and are projected onto the surface at high speed. As shown in Fig. 8a, in the Conventional Model, because the shot balls are discharged from the square exit of the control cage, they exhibit a roughly rectangular mass before the collision with the blade. Following the collision with the blade, as shown in Fig. 8b, the density of the shot balls is generally high in the central part and tends to decrease as it moves toward the edge. Therefore, when shot balls with spatial distributions are shot, the density of the shot marks is high in the central part of the substrate and tends to decrease toward the edge. Accordingly, the shot marks are concentrated in the center, and the coverage and uniformity deteriorate.

**Figure 9:**
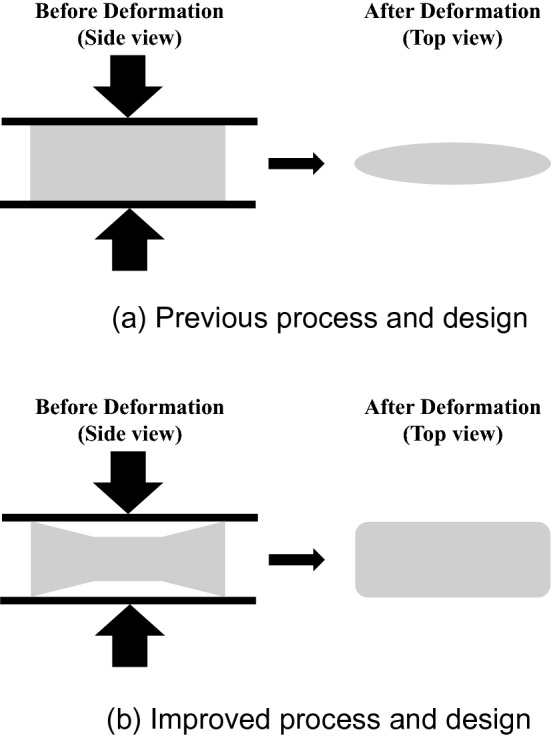
Improved design for hot plate rolling in the iron-making industry^26^.

The phenomenon of the distribution of elliptical-shaped shot marks when a square-shaped shot ball mass is pressed using a blade is similar to the phenomenon that occurs in a hot rolling process to manufacture a sheet metal by pressing a rectangular iron billet, as shown in Fig. 9^23,31^. In hot rolling of metal, this issue is resolved by realizing a new billet shape termed dog-bone rolling. Therefore, to improve coverage or uniformity, the shot ball mass discharged from the control cage should have a distribution with high density at the edge and low density at the center. With such a distribution, the coverage and uniformity of the substrate can be improved.

Therefore, in the Concave Model, in which the discharged outlet was changed to concave, the shot ball mass before the blade impact (Fig. 8c) was changed to a downward crescent shape, and that after the blade impact (Fig. 8d) was changed. The mass was slightly improved to a rectangular shape. Therefore, it is expected to exhibit an improved form of shot marks after collision with the substrate.

Similarly, in the Convex Model, in which the discharged outlet was changed to convex, the shot ball mass before the blade impact exhibited an upward crescent shape, which was contrary to that of the Concave Model, as shown in Fig. 8e. Therefore, as shown in Fig. 8f, following the collision with the blade, the shot ball mass is also changed to a square. Moreover, it is expected to exhibit an improved distribution of shot marks on the substrate as well.

### Shot mark distribution of models

**Figure 10:**
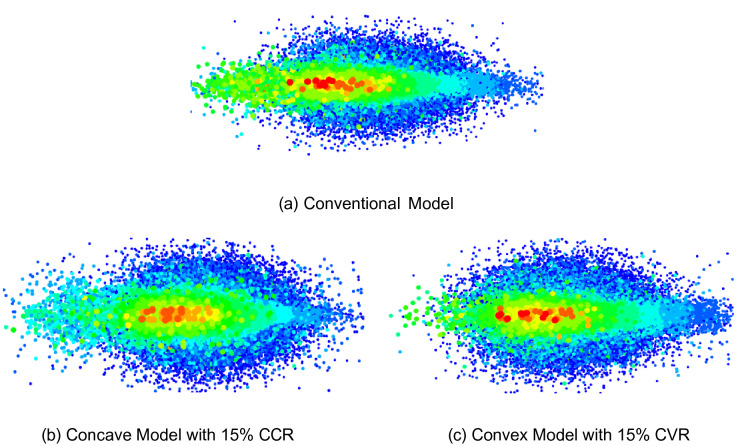
Shot mark distribution on substrate by Conventional, Concave, and Convex Models (Tecplot, https://www.tecplot.com/).

Figure 10a–c shows the distributions of shot marks in the Conventional, Concave, and Convex Models, respectively. The red color indicates a strong and large shot mark, and the blue color indicates a small shot mark. As expected, in the Conventional Model (Fig. 10a), significantly more shot balls are projected strongly in the center than at the edges. Moreover, the shot mark distribution exhibits a narrow and elongated elliptical shape. However, the shot mark distributions apply to the improved Concave Model with 15% CCR and the improved Convex Model with 15% CVR, as shown in Fig. 10b,c, respectively.

First, by comparing the Concave and Conventional Models, the width of the shot mark distribution in the Concave Model is less than that of the Conventional Model. Moreover, the Concave Model projects the shot balls significantly wider than the Conventional Model in the vertical direction. Similarly, the same phenomenon can be observed in the comparison of the Convex and Conventional Models. Therefore, compared with the case in the Conventional Model, in the Concave or Convex Model, the shot balls are distributed more uniformly on the substrate. Further, in the central area where green and red shot marks are concentrated, the Conventional Model exhibits a narrower elliptical shape (green and red) than the Concave or Convex Model. In other words, the distributions of the Concave and Convex Models have the form of a shorter ellipse (closer to a uniform distribution). Therefore, when applying the Concave and Convex Models, improvement in coverage or uniformity is qualitative. Therefore, the amount of improvement is also confirmed quantitatively in the following sections.

### Mass flow rate

**Figure 11:**
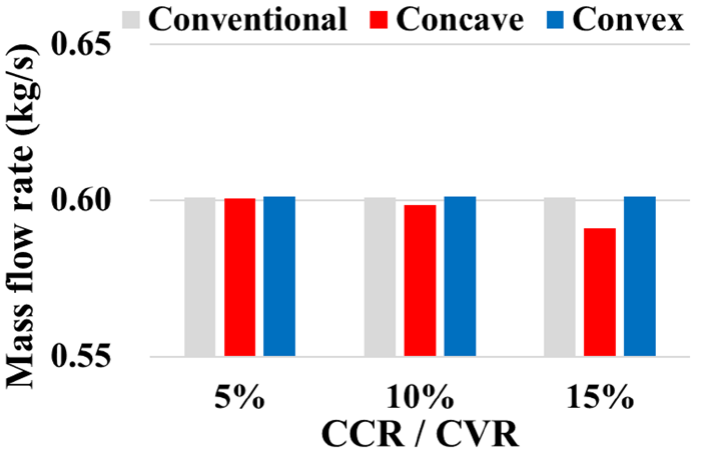
Mass flow rate according to models.

Generally, the number of shot balls use in shot blasting is significant, rendering the process expensive, and thereby cannot be ignored. Even if the same coverage is achieved, it is useful to consume a small number of shot balls. Therefore, the total number of shot balls consumed in the shot blaster should be controlled. First, for the Concave Model, the size of the hole through which shot balls are projected decreases as the concavity increases in the order of 5%, 10%, and 15% of the CCR. Therefore, the total mass of the consumed shot ball is inversely proportional to the CCR, as shown in Fig. 11. For the Convex Model, the larger the convexity, the larger the hole size. Moreover, the mass of the shot ball slightly increases as the CVR increases in the order of 5%, 10%, and 15%, as shown in Fig. 11. The reason for the smaller mass flow rate change of the Convex Model than that of the Concave Model is that the shape of the distributor does not change. Consequently, the Concave Model consumes fewer shot balls in the blaster than the Conventional Model, whereas the Convex Model consumes more shot balls than the Conventional Model. In other words, the Concave Model is the best in terms of economy.

### Coverage according to model type

**Figure 12:**
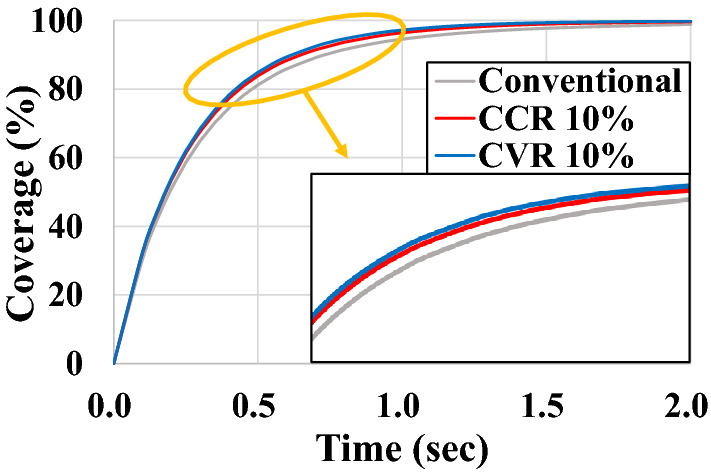
Coverage according to model type.

To confirm quantitative improvement using coverage, it is compared with the existing Conventional Model with rectangular holes, the Concave Model with the 10-% CCR, and the Convex Model with the 10-% CVR. As shown in Fig. 12, the coverages of all three models increase linearly up to approximately 0.2 s and reach saturation at approximately 1.0 s. Till approximately 0.2 s, as the number of shot marks in the shot ball is small, almost no overlapping area is observed between the shot balls. Therefore, the coverage increases linearly. Conversely, from approximately 1.0 s, the number of shot marks significantly increases and the area of ​​the shot marks overlapping each other also increases. Therefore, the slope of the coverage curve gradually decreases. The slope of the coverage curve for each model gradually increases in the order of the Conventional, Concave, and Convex Models. As expected, the coverage of the shot blaster using the Concave and Convex Models is higher than when using the Conventional Model, indicating that the efficiency of the shot blasting is increased. However, the difference between the Concave and Convex Models is insignificant, although the convex model exhibits a higher coverage.

### Coverage according to concave and convex ratios

**Figure 13:**
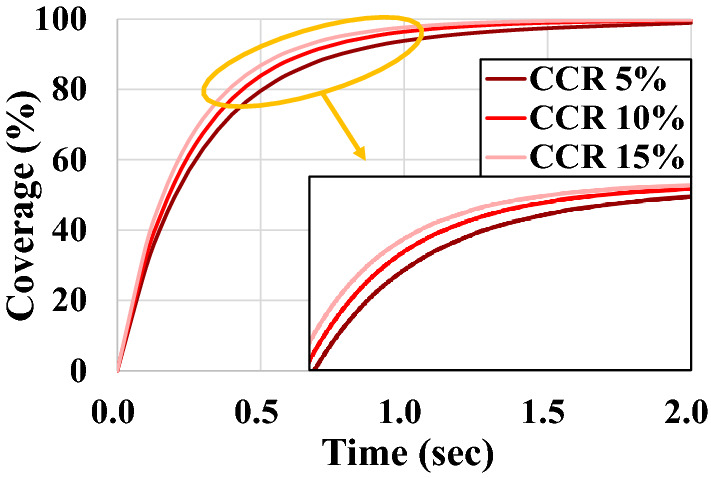
Coverage of concave models according to concave ratio.

First, the simulation was conducted by changing the Concave Model to a CCR of 5%, 10%, and 15% according to the degree of concavity. As shown in Fig. 13, all the models exhibit a general coverage curve shape. As the CCR increases, the coverage increases rapidly. In other words, the coverage is showing an effect of improving in proportion to the CCR. However, little improvement in the 5-% CCR is observed. Therefore, the coverage is similar to that of the Conventional Model.

**Figure 14:**
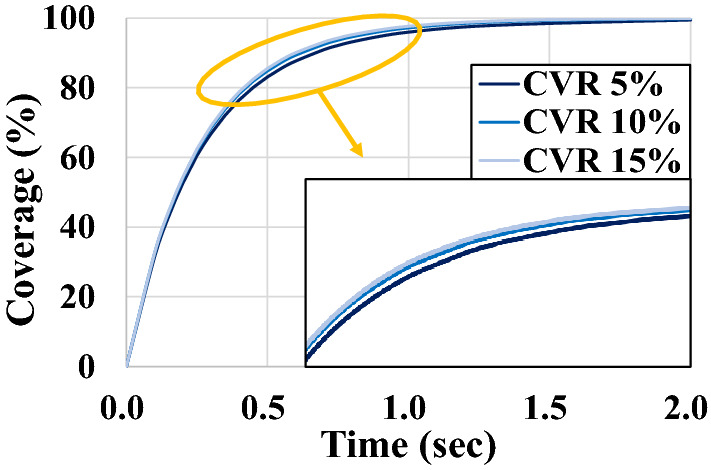
Coverage of convex models according to convex ratio.

Then, the coverage is measured for the Convex Model while changing the CVR to 5%, 10%, and 15%. The results are shown in Fig. 14. The Convex Model also exhibits a general coverage curve shape similar to that of the Concave Model, and the coverage is improved in proportion to the CVR. However, the overall degree of improvement does not vary significantly depending on the CVR. This is because the shape of the distributor is unchanged. Even if the hole size of the distributor is expanded, the number of discharged shot balls remains constant because the hole size of the distributor remains unchanged.

In the case of the 15-% CVR, both the coverage curves of the Convex and Concave Models are improved to a similar level. However, as the Convex Model uses several shot balls than the Concave Model, the Concave Model can be a better choice in terms of economy.

### Uniformity according to model type, concave ratio, and convex ratio

**Figure 15:**
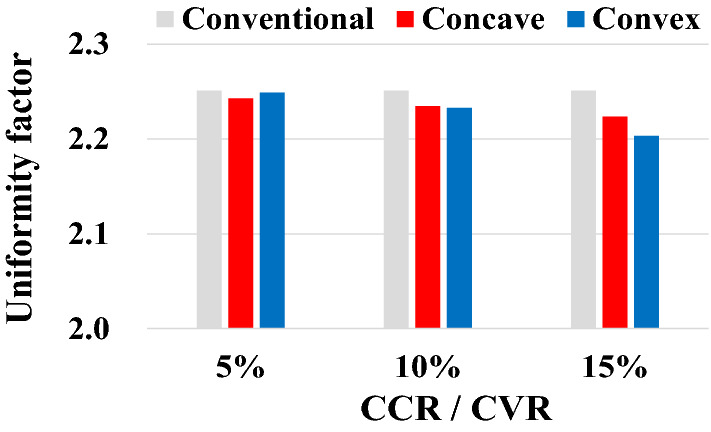
Uniformity of each model.

To increase the efficiency of mechanical impeller-type shot blasting, both coverage and uniformity must be improved. Therefore, the uniformities of the Concave and Convex Models are measured and compared with that of the Conventional Model. As shown in Fig. 15, the uniformities of all six models of the Concave and Convex Models are lower than that of the Conventional Model (2.251). In other words, the uniformity is improved, and the shot marks are more evenly distributed on the surface. Moreover, Fig. 15 shows that the dent marks are more evenly distributed as the CCR or CVR increases, regardless of the concave or convex shape. Among the Concave Models, the uniformity of the 15-% CCR has the smallest value (2.223), i.e., the most uniform shot mark. Notably, uniformity has the largest value in the order of 10-% and 5-% CCRs. Similarly, in the Convex Model, uniformity gradually decreases in the order of 5-%, 10-%, and 15-% CVRs. Therefore, when all seven models are considered, the 15-% Convex Model has the smallest uniformity, followed by the 15-% Concave Model.

### Experimental results

**Figure 16:**
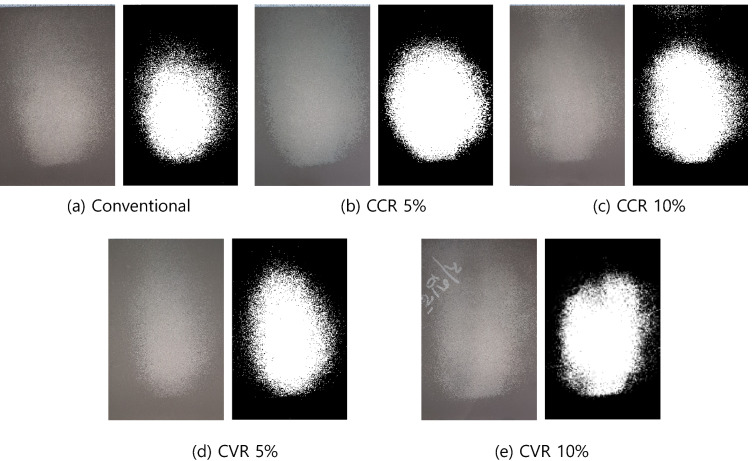
Shot ball marks for conventional, concave, and convex models.

The control cage of the conventional model, CCR 5%, CCR 10%, CVR 5%, and CVR 10% model was fastened to the blaster equipment, and shot blasting was performed. Fig. 16 shows shot marks that appeared when the shot balls collided on the surface of the specimen. First, compared with that corresponding to Fig. 16a, the CCR models corresponding to Fig. 16b,c have wider shot areas, that is, larger white areas. Similarly, in the CVR models corresponding to Fig. 16d,e, the shot area is increased compared with that of the conventional model. In other words, both the CCR and CVR models have increased shot areas compared with that of the conventional model.

**Figure 17:**
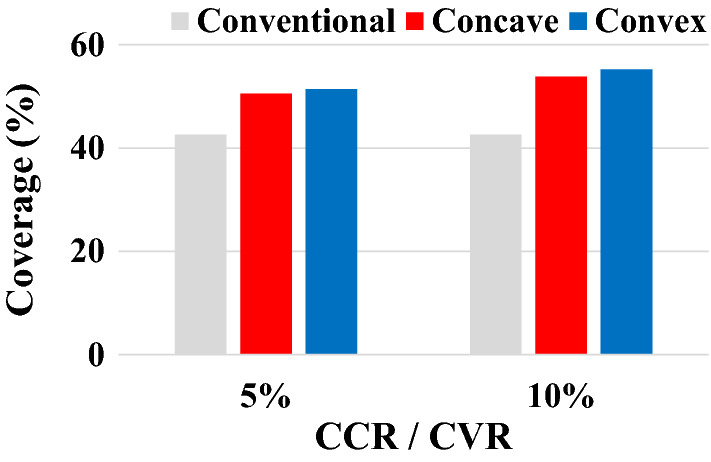
Coverage according to model type measured by experiments.

After image processing for the shot marks of each model in black and white colors, coverage was calculated. The results are shown in Fig. 17. As confirmed in the photo of the shot ball experiment shown in Fig. 16, coverage increases more significantly in both the CCR and CVR models than the conventional model. Moreover, it increases in proportion to the CCR and CVR. In other words, as the control cage is concave or convex, coverage increases. Although a quantitative difference exists between coverage and simulation, coverage is improved by changing the shape of the control cage hole to concave or convex.

## Conclusion

To evaluate the economics and efficiency of shot blasting, the model that is the most optimized from various viewpoints, such as coverage, uniformity, and the mass flow rate of shot balls, should be identified. First, in terms of coverage, the coverage increased faster in the Concave and Convex Models than in the Conventional Model. Moreover, in the Concave and Convex Models, coverage increased rapidly as the CCR and CVR increased, respectively. Second, in terms of uniformity, the Concave and Convex Models were superior to the Conventional Mode. Moreover, as CCR and CVR increased, they became superior. Third, in terms of mass flow rate, the Convex Model used more shot balls than the Conventional and Concave Models. Therefore, the Concave Model with a high CCR was the best. Moreover, in the experimental results, the Concave Model exhibited the best performance as well. Therefore, in this study, the newly proposed concave control cage model with a high CCR was the optimal model for shot blasting.

## Data Availability

The datasets generated during the current study are available from the corresponding author for reasonable requests.

## List of symbols

${A}_{s.m}$: Shot mark area of a shot ball
*R*: Radius of the shot ball
*δ*_*n*__*,ma*__*x*_: Maximum normal overlap distance
*L*: Length of a band
*d*: Width of a band
*C*: Coverage
CCR: Concave ratio
CVR: Convex ratio
$${H}_{cc}$$: Concave height
$${H}_{cv}$$: Convex height
${L}_{0}$: Length of the hole of the conventional control cage
